# Preliminary Comparative Analysis of Circulatory Glycosaminoglycan Concentrations and Disaccharide Profiles in Diabetic and Healthy Subjects

**DOI:** 10.5152/eurasianjmed.2026.251145

**Published:** 2026-06-24

**Authors:** Mohamad Warda, Mahmoud Gamal, Jiyuan Yang, Ke Xia, Fuming Zhang, Robert J. Linhardt

**Affiliations:** 1 Department of Biochemistry and Molecular Biology, Cairo University Faculty of Veterinary Medicine, Giza, Egypt; 2 Department of Physiology, Atatürk University Faculty of Veterinary Medicine, Erzurum, Türkiye; 3 Rensselaer Polytechnic Institute Center for Biotechnology and Interdisciplinary Studies, Troy, New York, USA; 4 Biotechnology Center and Departments of Chemistry and Chemical Biology, Rensselaer Polytechnic Institute Chemical and Biological Engineering and Biology, Troy, New York, USA

**Keywords:** Chondroitin sulfate, CS 2S6S, diabetes mellitus, glycosaminoglycans, LC, MS, MS

## Abstract

**Background:**

Type II diabetes (T2D) represents a significant global health challenge, necessitating deeper insights into its complex disease mechanism. While numerous efforts have advanced the understanding and treatment of T2D, the role of circulating glycosaminoglycans (GAGs) in diabetic plasma remains unclear. This study provides preliminary quantitative and qualitative screening of circulating GAG macromolecule levels in diabetic vs. healthy plasma.

**Methods:**

Plasma samples were collected from healthy and diabetic individuals (n = 12 per group). Then, via disaccharide composition liquid chromatography-tandem mass spectrometry (LC-MS/MS) analysis, the GAG species was quantified and their disaccharide profiles were evaluated.

**Results:**

Notably, a significant reduction in the relative abundance of the chondroitin sulfate (CS) 2S6S disaccharide was also observed (*P *= .004). Additionally, CS concentrations in healthy human plasma were approximately 70 and 100 times higher than those of hyaluronic acid (HA) and heparan sulfate (HS), respectively, highlighting differential enzymatic or nonenzymatic turnover rates among GAG species. Interestingly, circulating CS, HS, and HA levels remained stable in diabetic plasma.

**Conclusion:**

These findings elucidate the downregulation of a specific disaccharide variant (CS 2S6S) as a potential biomarker of T2D. Further research is warranted to elucidate the mechanistic underpinnings of these observations and their clinical implications.

**Main Points**

- Plasma glycosaminoglycans (GAGs) show measurable alterations in individuals with type II diabetes (T2D).
- Chondroitin sulfate (CS) levels are significantly elevated in T2D plasma, while heparan sulfate and hyaluronic acid remain largely unchanged.
- Disaccharide profiling reveals specific compositional shifts, including a reduction in 2S6SCS abundance in diabetic plasma.
- These GAG alterations are consistent with extracellular matrix remodeling and may contribute to diabetes-related complications.
- Plasma GAGs hold potential as biomarkers and therapeutic targets in T2D, warranting validation in larger, diverse cohorts.

## Introduction

Glycosaminoglycans (GAGs), the glycan components of proteoglycans, protect protein surfaces and mediate cell communication, showing organ-specific diversity in structure and sulfation.^1^^,^^2^ Their structural diversity underlies key roles in development, signaling, cell migration, and responses to inflammation and viral infection.^1^^,^^3^ Glycosaminoglycans act as regulators of cytokines, chemokines, growth factors, and enzymes, undergoing dynamic synthesis and modification that can influence disease development. The 4 main GAG classes are heparin/heparan sulfate (HS), chondroitin sulfate (CS)/dermatan sulfate (DS), keratan sulfate, and hyaluronic acid (HA). Chondroitin sulfates consist of repeating disaccharides of N-acetyl-D-galactosamine (GalNAc) with D-glucuronic (GlcA) or L-iduronic acid (IdoA), whereas HSs are composed of N-acetyl- or N-sulfated-D-glucosamine (GlcNAc/GlcNS) with GlcA or IdoA.^2^

Changes in sulfation patterns have been implicated in the development of various diseases, including complications associated with diabetes.^4^ For example, alterations in heparan sulfate structures within the glomeruli have been linked to diabetic nephropathy, suggesting a role for sulfation modifications in kidney dysfunction associated with diabetes.^5^ Dysregulation of sulfur-metabolizing enzymes has been observed in diabetic nephropathy, suggesting that impaired sulfation pathways may contribute to renal complications in diabetes.^6^ Altered glycosaminoglycan sulfation patterns are linked to extracellular matrix remodeling and endocrine dysfunction in pancreatic islet cells, contributing to type 2 diabetes (T2D) progression.^7^ These findings highlight the importance of elucidating sulfation dynamics in diabetic pathophysiology.

Chondroitin sulfate, on the other hand, is a predominant GAG with essential physiological and pathological roles in animals.^8^ The 4 main CS subtypes—CS-A, CS-C, CS-D, and CS-E—are distinguished by unique sulfation patterns: CS-A has 4-O-sulfated GalNAc, CS-C has 6-O-sulfation, CS-D contains 2-O-sulfated GlcA, and CS-E features 4,6-O-disulfated GalNAc.^9^

Besides these well-known subtypes, CS-O is a major circulating isomer with variable sulfation patterns that underlie its functional diversity.^10^ This study highlights the importance of CS-O, a nonsulfated chondroitin, in circulation and its potential biological roles. Both sulfated and nonsulfated CS influence human colonic microbiota composition and metabolism, indicating significant interactions with gut bacteria.^11^ Further studies emphasize the role of nonsulfated chondroitin in tissue remodeling, showing its capacity to modulate the extracellular matrix, promote cell migration, and support tissue repair.^12^ These findings highlight the potential therapeutic role of nonsulfated chondroitin in wound healing and tissue regeneration. Minor CS isomers, including 2-O,4-O-disulfated chondroitin sulfate (2S4S-CS) and chondroitin sulfate trisulfate (CS-tri), have also been detected in various biological fluids.^10^ Although present in low abundance, these minor isomers are believed to play key roles in modulating extracellular matrix interactions and cellular signaling. Sulfation of GAGs is essential for normal physiology. The previous mouse study showed organ-specific HS sulfation, with lower levels in kidney and intestine than in liver and brain.^13^ Altered GAG sulfation contributes to disease, including diabetic complications, by affecting extracellular matrix remodeling, pancreatic islet function, and HS-mediated signaling, adhesion, and growth factor interactions that regulate development, wound healing, and inflammation.^14^

In contrast, HA—the only unsulfated GAG—is synthesized by membrane enzymes and rapidly extruded as a continuously elongating disaccharide chain.^15^ Hyaluronic acid, a high-molecular-mass (>1 000 kDa) extracellular matrix (ECM) GAG composed of repeating GlcNAc–GlcA disaccharides, regulates tissue hydration, elastoviscosity, proteoglycan assembly, and receptor-mediated processes including cell attachment, migration, wound healing, tumorigenesis, and inflammation.^16^

**Figure 1.**
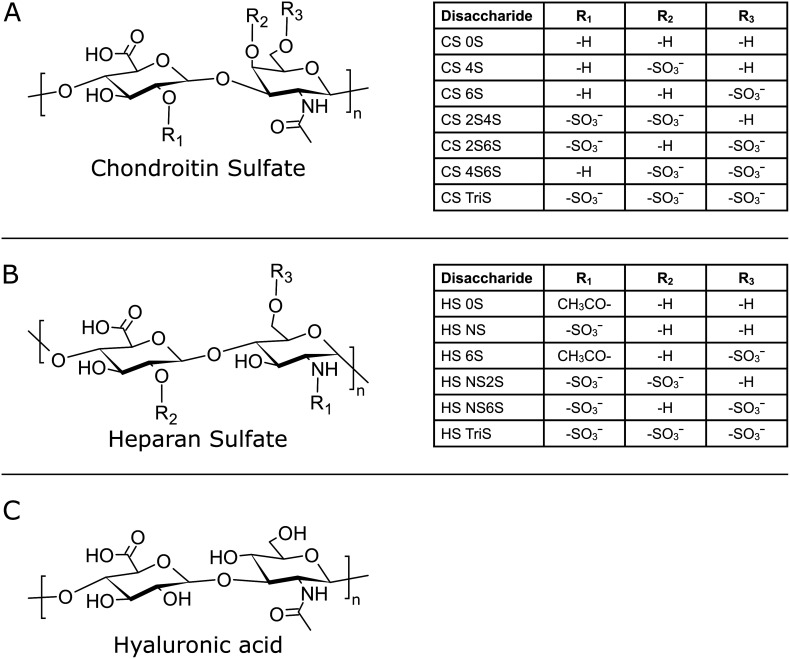
The chemical structure of the analyzed glycosaminoglycans. (A) Chondroitin sulfate (CS) is a polymer of the repeating disaccharide: (1-4)-β-glucuronic acid and (1-3)-β-N-acetyl-d-galactosamine. Chondroitin sulfate has 3 positions available for sulfation: 2-O-sulfation on GlcA (R_1_), 4-O-sulfation on GalNAc (R_2_), and 6-O-sulfation on GalNAc (R_3_). (B) Heparan sulfate (HS) is a polymer of the repeating disaccharide: (1-4)-β-glucuronic/iduronic acid and (1-4)-α-d-glucosamine. Heparan sulfate has 3 positions available for sulfation: N-sulfation on GlcN (R_1_), 2-O-sulfation on GlcA (R_2_), and 6-O-sulfation on GlcN (R_3_). (C) Hyaluronic acid (HA) is a polymer of the repeating disaccharide: (1-4)-β-glucuronic acid and (1-3)-β-N-acetyl-d-glucosamine.

Glycosaminoglycans extending into the vessel lumen protect endothelial cells from blood flow, sense shear stress, and regulate charge-dependent vascular permeability. They are continuously degraded and renewed by shearing forces and enzymes like heparanase and hyaluronidase.^9^ This imbalance may drive diabetic vasculopathies, with glycocalyx remodeling potentially aiding non–insulin-dependent glucose clearance.^17^ Assessing GAG levels in circulation could provide insights into their accelerated tissue degradation observed in T2D.^18^ Recent investigations have focused primarily on whole GAG species rather than on their quantitative and qualitative disaccharide compositions.^19^ Other studies have attempted to resolve the disaccharide compositions of the recovered GAG species in normal plasma samples.^10^ This study aimed to characterize the distribution of GAG species (CS, HS, and HA) and their disaccharide compositions in diabetic plasma compared with normal controls. The chemical structures of the analyzed GAGs and the assessed disaccharides are illustrated in Figure 1.

## Material and Methods

### Plasma Samples

Plasma was obtained from 12 male patients with a documented history of uncontrolled T2D (mean age 52.6 ± 7.6 years) and from 12 age-matched healthy male volunteers (mean age 54.4 ± 8.4 years). All participants provided written informed consent prior to sample collection. Collection and use of plasma adhered to the ethical standards of the Declaration of Helsinki and to institutional guidelines for research involving human-derived materials. The study was approved by the Ethics committee of Cairo University (Approval no: CU-MED-REC-2026-0429 Date: April 29, 2026). All samples were anonymized, and no personal data were collected. Blood tests confirmed uncontrolled diabetes and excluded other complications (Table 2). Diabetic patients had persistently Albustix-positive urine but no renal or hepatic issues. Plasma samples (500 µL) from diabetic and healthy individuals were lyophilized for GAG analysis.

### Chemicals

Unsaturated disaccharide standards of CS (ΔUA-GalNAc; ΔUA-GalNAc4S; ΔUA-GalNAc6S; ΔUA2S-GalNAc; ΔUA2S-Gal-NAc4S; ΔUA2S-GalNAc6S; ΔUA-GalNAc4S6S; ΔUA2S-GalNAc4S6S), unsaturated disaccharide standards of HS (ΔUA-GlcNAc; ΔUA-GlcNS; ΔUA-GlcNAc6S; ΔUA2S-GlcNAc; ΔUA-GlcNS; ΔUA-GlcNS6S; ΔUA2S-GlcNAc6S; ΔUA2S-GlcNS6S), and the unsaturated disaccharide standard of HA (ΔUA-GlcNAc), where ΔUA is 4-deoxy-α-L-threo-hex-4-enopyranosyluronic acid, were purchased from Iduron (Manchester, UK). Actinase E was obtained from Kaken Biochemicals (Tokyo, Japan). *Escherichia coli* expression and purification of the recombinant *Proteus vulgaris* chondroitin lyase ABC were performed in the laboratory. Recombinant Flavobacterial heparin lyases I, II, and III were expressed in the laboratory via *E. coli* strains. 2-Aminoacridone (AMAC) and sodium cyanoborohydride were obtained from Sigma-Aldrich (St. Louis, MO, USA). All the solvents used were of high-performance liquid chromatography (HPLC) grade.

### Extraction and Digestion of Glycosaminoglycans

Freeze-dried plasma samples were reconstituted and proteolyzed at 55°C for 24 hours with Actinase E (400 µL, 25 mg/mL), then deactivated at 100°C for 30 minutes. The digest was transferred to a 10 kDa MWCO spin tube, mixed with 2% CHAPS, and washed. Digestion buffer (300 µL, 50 mM ammonium acetate, 2 mM CaCl_2_, pH 7.0) containing heparin lyases I–III and chondroitin lyase ABC (10 mU each) was added, and samples were incubated at 37°C for 24 hours. Digestion was terminated by ultrafiltration; filtrates were washed, combined, and freeze-dried to yield disaccharides.

### Derivatization of Unsaturated Disaccharides with Aminoacridone-Labeling

The dried samples were AMAC labeled as described earlier^20^ by adding 10 μL of 0.1 M AMAC in DMSO/acetic acid (17/3, *v/v*) and incubating at room temperature for 10 minutes, followed by the addition of 10 μL of 1 M aqueous sodium cyanoborohydride and incubation for 1 hour at 45°C. A mixture containing all 17 disaccharide standards prepared at 0.5 ng/μL was similarly AMAC labeled and used for each run as an external standard. After the AMAC-labeling reaction, the samples were centrifuged, and each supernatant was recovered.

### LC-MS/MS

LC was performed on an Agilent 1200 system (Agilent Technologies) at 45°C using a Poroshell 120 ECC18 column (2.7 μm, 3.0 × 50 mm). Mobile phases were 50 mM ammonium acetate (A) and methanol (B) at 300 μL/min. The gradient was 0-10 minutes, 5%-45% B; 10-10.2 minutes, 45%-100% B; 10.2-14 minutes, 100% B; 14-22 minutes, 100%-5% B, with a 5 μL injection volume. A triple quadrupole MS with electrospray ionization (ESI) (Thermo Fisher Scientific) was used in multiple reaction monitoring (MRM) mode, adapted from Linhardt et al.^21^ MS was operated in negative ionization mode with a spray voltage of 3000 V, vaporizer temperature of 400°C, and capillary temperature of 250°C. A scheduled MRM method divided the LC run into 3 segments (0.0-6.5, 4.5-13.5, 6.5-13.5 minutes) based on compound elution. This targeted approach monitored only relevant transitions, increasing dwell time, sensitivity, and peak definition while reducing noise. Each segment included transitions for AMAC-labeled disaccharides (Table 3).

### MS Quantitative Analysis

The concentration of the target disaccharides in the plasma samples was determined via relative quantification, in which the chromatographic peak area of the sample (*A*_sample_) was compared with that of an independently analyzed standard (*A*_standard_) under the same conditions, followed by multiplication by the standard concentration (*C*_standard_). The calculation follows the equation:

*C*_ sample_ = (𝐴_sample_/𝐴_standard_) * 𝐶_standard_.

This method corrects for sulfation-induced ionization efficiency differences by standardizing analytical conditions and directly comparing peak areas.

### Statistical Analyses

The total concentrations of various GAGs in healthy and diabetic individuals were compared using 2-way ANOVA. Assumptions were verified with the Shapiro–Wilk test for normality and Levene’s test for homoscedasticity on log10-transformed data. Bonferroni correction was applied for multiple comparisons. Individual GAGs, their disaccharides, and blood chemistry parameters were compared between diabetic and control groups using Student’s *t*-test after validating assumptions as above. All statistical analyses and figure generation were performed in RStudio (v2023.06.1-524) using R (v4.3.1).^22^^,^^23^ The data are expressed as the means ± SDs. Differences were considered significant at *P* < .05.

This exploratory study did not include an a priori power calculation because no plasma GAG disaccharide data exist to estimate effect sizes or variance in diabetic versus healthy individuals. Sample size was instead guided by prior methodological work, such as Han et al (Glycobiology, 2020), who used 6 samples; 12 per group were used to provide a more conservative exploratory design.

## Results

### Glycosaminoglycans Analysis via LC-MS/MS

**Figure 2.**
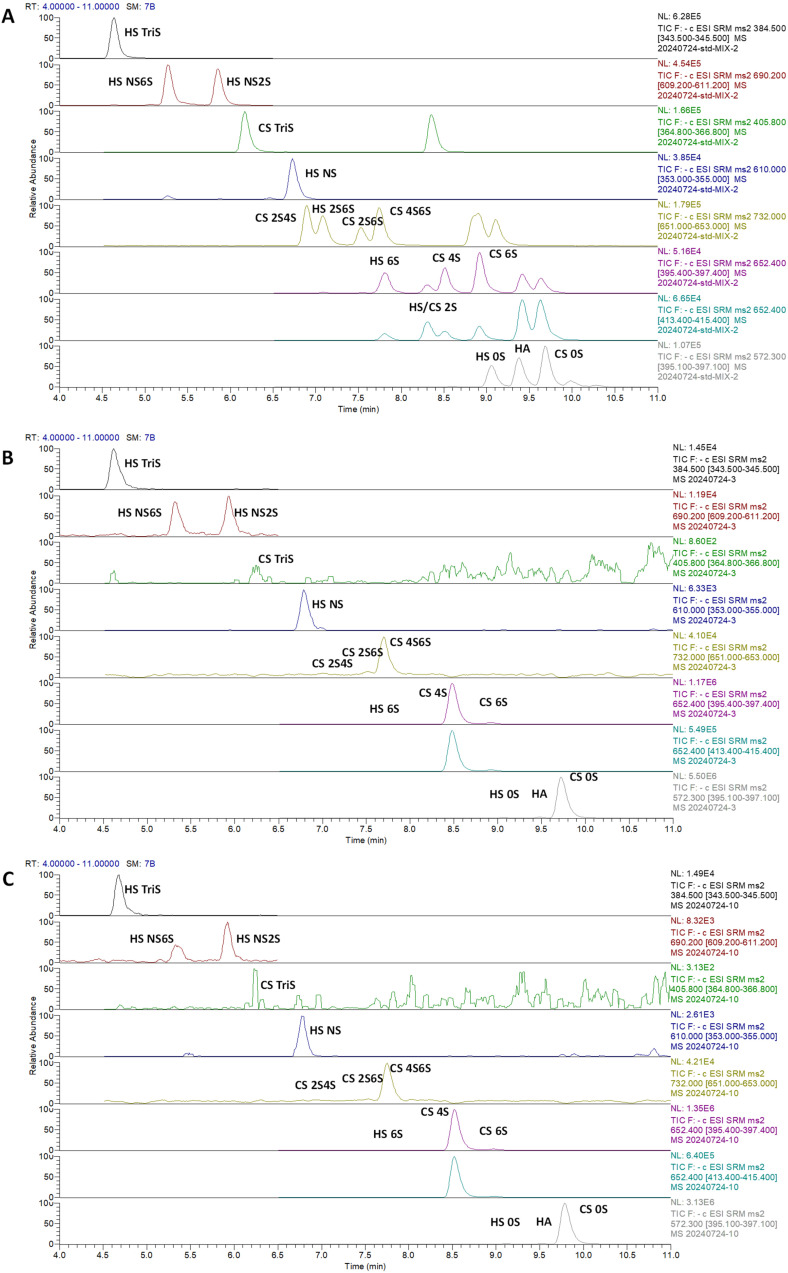
Extracted ion chromatograms (EICs) of AMAC-labeled disaccharides. (A) Analysis of AMAC-labeled disaccharide standards, with assigned peaks used to determine the disaccharide composition of the samples. (B) AMAC-labeled disaccharides from a healthy plasma sample after heparin lyase treatment. (C) AMAC-labeled disaccharides from a diabetic plasma sample after heparin lyase treatment. The small, unlabeled peaks in the third EIC are noise due to the extremely low concentration of the CS TriS.

The analysis of disaccharide units of GAGs in biological samples via the LC–MS/MS method previously established is effective for separating and quantifying disaccharide components.^21^ Samples were digested with heparin lyases I–III and chondroitin lyase ABC to release GAG disaccharides, which were AMAC-labeled via reductive amination for improved resolution. Labeled disaccharides were analyzed by reversed-phase LC–MS/MS, displayed as extracted ion chromatograms. Figure 2A shows standards for peak identification and quantification, while Figures 2B and C show healthy and diabetic plasma profiles, respectively. Distinct peaks enabled comparative GAG quantification, with some weaker plasma signals indicating lower disaccharide levels. Quantification was performed by integrating peak areas relative to the standards.

### Glycosaminoglycans Content and Composition in Human Plasma

**Figure 3.**
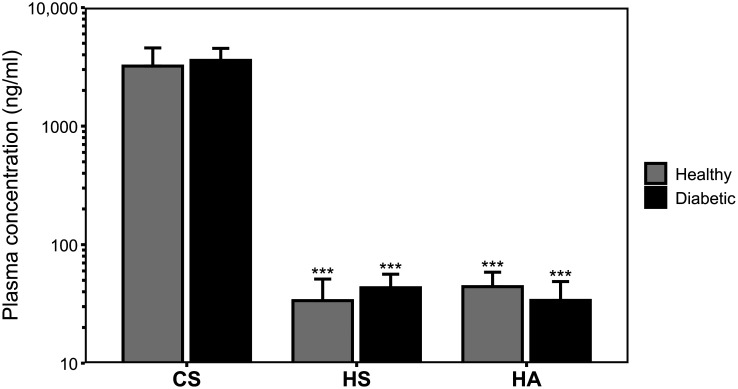
Glycosaminoglycans composition in healthy and diabetic human plasma. The data were analyzed via 2-way analysis of variance and are presented as the means ± SDs. ***P < .001 compared with the chondroitin sulfate of healthy individuals.

The concentrations of each GAG in the plasma of healthy and diabetic subjects are shown in Figure 3. The plasma concentration of CS in healthy individuals (3.21 ± 1.36 μg/mL) was several-fold greater than those of HS (33.7 ± 17.6 ng/mL) and HA (44.2 ± 14.3 ng/mL). The concentrations of CS, HS, HA, and total GAGs in healthy individuals did not differ significantly from their concentrations in diabetic subjects, as shown in Table 1.

**Figure 4.**
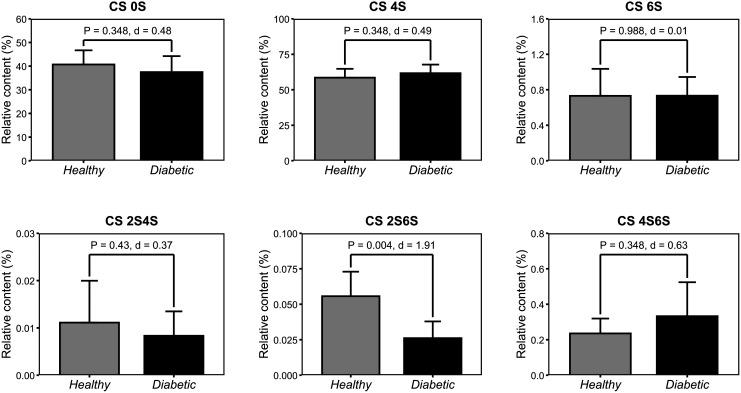
Relative content of CS disaccharides in the healthy and diabetic groups. The data were analyzed via Student’s *t*-test and are presented as the means ± SDs. Differences were considered significant at *P* < .05.

**Figure 5.**
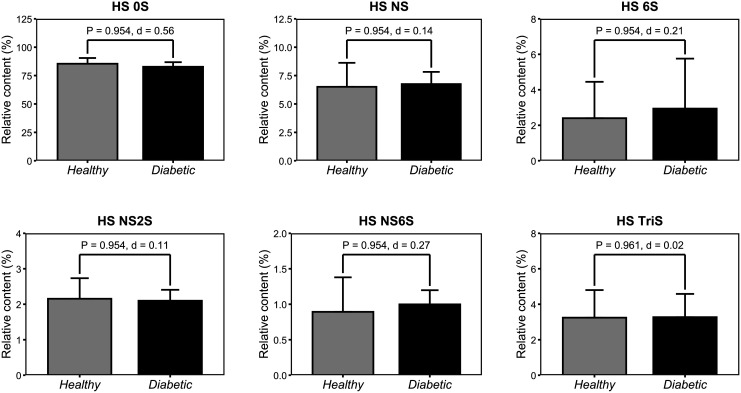
Relative content of HS disaccharides in the healthy and diabetic groups. The data were analyzed via Student’s *t*-test and are presented as the means ± SDs. Differences were considered significant at *P* < .05.

The relative concentrations of CS and HS disaccharides are shown in Figures 4 and 5, respectively. Only the relative content of 2S6SCS was significantly greater in the healthy group (0.056% ± 0.017%) than in the diabetic group (0.026% ± 0.012%). However, the other CS and HS disaccharides did not significantly differ.

## Discussion

Over recent decades, T2D has become a major global health challenge due to its complex nature and wide range of systemic complications.^18^ Glycosaminoglycans, major constituents of the ECM and glycocalyx, play crucial roles in regulating cellular functions, including endocrine pancreatic activity, and are implicated in the pathophysiology of T2D. They typically exert a scaffold-like protective effect against cellular insults, such as oxidative stress in β-cells.^24^^,25^ Given the toxicity of hyperglycemia in diabetes to the ECM and the glycocalyx,^26^ early research assessed the extent of GAG sulfation in diabetes.^27^ Later investigations focused primarily on the quantification of GAG concentrations rather than the qualitative analysis of their disaccharide composition during diabetes.^19^ Although GAG disaccharide alterations in diabetes remain poorly understood, investigating compositional changes in diabetic plasma is essential. This study is the first to quantify individual GAG species and characterize disaccharide components of depolymerized plasma GAGs, providing insights into their potential role in T2D. Measured concentrations of total CS, 4SCS, and 0SCS in normal plasma closely matched reference ranges reported by Bratulic et al,^10^ the novel work distinguishes itself by presenting the first-ever documentation of normal values for recovered HS in plasma and its disaccharide fractions, both in normal and diabetic plasma. This groundbreaking study extends beyond previous research by extending the scope to include HS and its disaccharide fractions (e.g., 0SHS and NSHS) in plasma.

Furthermore, several in vitro experiments have demonstrated the contribution of GAGs, particularly HSs and CSs, to the formation of amyloid fibrils through their anionic component-aided scaffolding of various proteins.^28^ A previous study reported that HSs, followed by HAs, are the primary GAGs involved in aggregating lipid-free mouse plasma amyloids into defined fibrils, whereas monosulfated CSs form spherical particles with short protofibrils.^29^ Altered CS metabolism is linked to inflammation, coagulation, and cell signaling. Chondroitin sulfate modulates insulin signaling and glucose homeostasis through interactions with growth factors and cytokines. The markedly reduced plasma 2S6SCS in diabetic patients suggests disrupted metabolism or turnover of specific CS disaccharides, consistent with previous reports of decreased 2S6SCS in diabetic rat brains.^30^ This underscores the context-dependent regulation of CS metabolism in diabetes, likely influenced by tissue-specific enzymatic remodeling, altered sulfotransferase expression, and extracellular matrix dynamics. The biosynthesis of 2S6SCS relies on the coordinated activity of chondroitin 2-O-sulfotransferase and chondroitin 6-O-sulfotransferase, which may be disrupted under hyperglycemic conditions.^28^ The reduction of 2S6SCS in plasma suggests enhanced enzymatic degradation, possibly due to increased chondroitinase activity or altered clearance mechanisms in diabetes. As CS sulfation is crucial for cell signaling, inflammation, and endothelial integrity, the observed loss of 2S6SCS may contribute to vascular dysfunction and diabetic complications. Since plasma GAGs primarily originate from vascular shedding and systemic ECM remodeling, endothelial surfaces and highly permeable tissues such as the kidney and liver are likely the main sources of this altered sulfation pattern. The interplay among vascular permeability, enzymatic remodeling, and metabolic shifts in diabetes warrants further investigation. Although this study did not directly assess the relationship between diabetes severity and CS composition, previous studies have shown that CS modifications influence diabetic complications and tissue remodeling.^31^ Notably, significant alterations in CS quantity and sulfation patterns have been observed in disease states, including increased CS levels in tumor tissues compared with adjacent normal tissues;^32^ this underscores the potential role of CS in pathological tissue remodeling. In diabetic kidneys, a pronounced reduction in 4-O-sulfated disaccharides, accompanied by decreased expression of chondroitin 4-O-sulfotransferase, has been documented, suggesting that disruptions in GAG sulfation patterns may contribute to diabetes-related complications.^28^ Similarly, the observed decrease in 2S6SCS disaccharide levels in diabetic plasma may reflect disruptions in chondroitin sulfotransferase activity.^33^ This is particularly evident in enzymes responsible for 2-O and 6-O sulfation. Dysregulation of these enzymes has been associated with prolonged hyperglycemia and diabetes severity, resulting in structural modifications of GAGs. Future longitudinal studies examining diabetes progression and its effects on CS enzymatic regulation will be essential to clarify these mechanisms. Moreover, investigating the relationship between glycemic control and CS disaccharide composition may help identify molecular markers for assessing diabetes severity and related complications.

The previous analysis of placental GAG modulatory enzymes in preeclampsia demonstrated significant changes in GAG sulfation patterns.^13^ This finding suggests a shared underlying mechanism contributing to altered sulfated GAG profiles in both diabetic and preeclampsia patients. The specific reduction of the 2S6SCS disaccharide in diabetic plasma may serve as a potential molecular marker for elucidating diabetes-associated pathophysiology, warranting further investigation into its precise roles and mechanisms. Moreover, the observed variation in CS disaccharide patterns across different body fluids is particularly noteworthy. While a previous study revealed a high percentage of 6-sulfated disaccharides in the urine of diabetic nephropathy patients,^34^ the findings show comparable CS levels in both diabetic and control plasma. This discrepancy suggests differential compartmentalization of CS disaccharides between plasma and urine in diabetic patients, potentially reflecting enhanced shedding of 6-sulfated CS during diabetic nephropathy rather than simple filtration from the bloodstream.

The lack of a significant difference in total CS levels between healthy and diabetic groups suggests that diabetes-related disturbances do not affect overall CS production or turnover but rather alter its sulfation pattern. Moreover, the relative abundance of 2S6SCS is low (0.056% ± 0.017%), indicating that changes in this disaccharide may be compensated without substantially impacting the distribution of other CS components, as observed in this study.

Moreover, the absence of any significant difference in the plasma concentrations of HS and HA between the healthy and diabetic groups in the study implies that these GAGs are not affected by diabetic conditions. Since HA plays a role in tissue hydration and lubrication, its stable level might indicate that the pathways regulating HS and HA are not disrupted in diabetes or that compensatory mechanisms maintain their homeostasis. This observation contradicts previous research findings that indicated a significant increase in HA levels during hyperglycemia, as documented by Nieuwdorp et al.^35^ This discrepancy may reflect limitations of their enzyme-linked immunosorbent assay (ELISA) method, which detects only certain hyaluronan lengths rather than assessing all HA categories, as done in the approach.

### Study Strengths and Limitations

This study provides the first comprehensive quantitative and qualitative characterization of circulating GAGs in the plasma of individuals with T2D, offering valuable insights into their structural modifications. The use of enzymatic depolymerization combined with advanced analytical techniques, including AMAC labeling and LC–MS/MS, enabled detailed disaccharide profiling and improved understanding of GAG composition in diabetic plasma. The results link GAG alterations to diabetes-related complications such as impaired wound healing, inflammation, and vascular dysfunction, thereby contributing to a deeper understanding of diabetes pathophysiology. Notably, the marked reduction in plasma 2S6SCS concentration in diabetic patients suggests its potential as a molecular marker for diagnostic or prognostic applications.

This study has certain limitations. As a preliminary investigation, it offers initial insights into circulatory GAG alterations in diabetes; however, larger and more diverse cohorts are required to validate and generalize these findings. Although differences in GAG profiles were observed, interpreting these alterations in terms of causation or their role in diabetes-related complications remains challenging. Further research is also needed to correlate CS and HS disaccharide changes with disease severity and progression.

Another limitation is that no a priori power analysis was performed because effect-size estimates for plasma GAG disaccharides in diabetes were not available. The sample size therefore followed prior exploratory GAG studies and used 12 well-characterized plasma samples per group. The effect sizes identified here—particularly for 2S6S-CS—now offer the first data needed to inform power calculations in future studies.

This study represents a comprehensive exploration of different GAG species recovered from diabetic plasma, shedding light on their disaccharide compositions compared with those of normal control plasma. The recorded changes in the sulfation patterns of CS address the complexity of CS sulfation and its potential impact on cellular interactions and signaling during diabetes. These findings emphasize the altered expression of chondroitin sulfotransferases, highlighting complex modifications in chondroitin sulfate variants during diabetes. Additionally, the stable plasma circulating levels of both HS and HA indicate that compensatory mechanisms may maintain their homeostasis.

## Figures and Tables

**Table 1.** The Plasma Concentrations of Total and Specific GAGs in the Healthy and Diabetic Groups

**Parameter**	**Healthy**	**Diabetic**	***P* ^a^**
CS (μg/mL)	3.21 ± 1.36	3.59 ± 0.94	.44
HS (ng/mL)	33.7 ± 17.6	43.3 ± 13	.59
HA (ng/mL)	44.2 ± 14.3	33.8 ± 15	.097
Total GAGs (μg/mL)	3.29 ± 1.37	3.66 ± 0.97	.3

^a^The data were analyzed via Student’s *t*-test. Differences were considered significant at *P* < .05.

CS, chondroitin sulfate; GAGs, glycosaminoglycans; HA, hyaluronic acid; HS, heparan sulfate.

**Table 2.** Blood Chemistry Profiles for 2 Groups of 12 Male Subjects Each with Normal and Uncontrolled Diabetes (Without Renal or Hepatic Complications)

**Parameters**	**Normal Group** **(Mean ± SD)**	**Uncontrolled Diabetes Group** **(Mean ± SD)**	***P***
Age (years)	54.4 ± 8.4	52.6 ± 7.6	.581
Systolic blood pressure (mmHg)^a^	122.8 ± 15.9	139.8 ± 13	.009
Diastolic blood pressure (mmHg)^a^	75 ± 8.37	91.75 ± 9.13	<.001
Fasting glucose (mg/dL)^b^	83.9 ± 9.9	212.8 ± 37.5	<.001
Hemoglobin A1C (HbA1c,%)^b^	5.16 ± 0.18	9.8 ± 1.41	<.001
Total cholesterol (mg/dL)^c^	185.2 ± 32.7	218.5 ± 70.5	.089
Low-density lipoprotein-cholesterol (LDL-C) (mg/dL)^c^	102.8 ± 23.1	144.6 ± 13.7	<.001
High-density lipoprotein-cholesterol (HDL-C) (mg/dL)^c^	50.1 ± 7.2	46.8 ± 11.2	.4
Triglycerides (mg/dL)^c^	120.6 ± 25	170 ± 23.9	<.001
Creatinine (mg/dL)^d^	1.03 ± 0.12	1.05 ± 0.11	.725
Alanine transaminase (ALT) (U/L)^d^	25.3 ± 8.77	29.55 ± 7.33	.211
Aspartate transaminase (AST) (U/L)^d^	21.27 ± 7.57	22.31 ± 9	.763
Albumin (g/dL)^d^	4.39 ± 0.46	4.43 ± 0.21	.768

^a^Blood pressure: Expressed as systolic/diastolic values with standard deviations for each.

^b^Fasting glucose and HbA1c: Clearly, distinguishing normal glycemic levels from those typical of uncontrolled diabetes.

^c^Lipid profile: highlights differences between the groups, showing typical dyslipidemia in patients with uncontrolled diabetes.

^d^Liver and renal markers: Parameters such as creatinine, ALT, and AST are within normal ranges to ensure the absence of renal or hepatic complications.

**Table 3.** MRM Transitions and Corresponding Time Segments

**Segment**	**Time Window (minutes)**	**Precursor Ion (*m/z*)**	**Product Ion (*m/z*)**
Segment 1	0.0-6.5	690.200	609.200-611.200
		384.500	343.500-345.500
Segment 2	4.5-13.5	610.000	353.000-355.000
		732.000	651.000-653.000
		405.800	364.800-366.800
Segment 3	6.5-13.5	652.400	395.400-397.400
		652.400	413.400-415.400
		572.300	395.100-397.100

## Data Availability

The data that support the findings of this study are available on request from the corresponding author.
